# Supplementary material for: Distinct Cytokine and Cytokine Receptor Expression Patterns Characterize Different Forms of Myositis
Source: medRxiv. 2025 Feb 21:2025.02.17.25321047. Preprint. [Version 1] doi: 10.1101/2025.02.17.25321047 (PMC11875321; doi:10.1101/2025.02.17.25321047)

Supplementary Methods.

Patients

Muscle tissue from 669 patients who underwent diagnostic muscle biopsies and healthy volunteers at several centers specialized in neuromuscular diseases underwent bulk RNA sequencing (RNA-seq) analysis. Muscle biopsies from myopathy patients were obtained from institutional review board-approved longitudinal cohorts from the National Institutes of Health in Bethesda, MD, the Johns Hopkins Myositis Center in Baltimore, MD, the Vall d'Hebron and Clinic Hospitals in Barcelona, Spain, the Mayo Clinic in Rochester, MN, and the Charité-Universitätsmedizin in Berlin, Germany.

All samples were divided into clinical groups, including DM, IMNM, ASyS, IBM, overlap myositis, other inflammatory myopathies, genetic myopathies, and normal tissue (NT). Some samples were also classified into autoantibody subgroups, including Mi2, MDA5, NXP2, TIF1, HMGCR, SRP, Jo1, and PM/Scl (Table 1). Since the only overlap myositis samples analyzed were from anti-PM/Scl patients, we will refer to PM/Scl as both a clinical group and an autoantibody subgroup, in order to compare it to other myopathies from both categories. Patients were classified as IBM if they fulfilled Lloyd's criteria for inclusion body myositis[1]. Patients testing positive for a myositis-specific autoantibody were classified according to the Casal and Pinal criteria[2]. The same criteria were used to classify patients as PM/Scl, although anti-PM/Scl autoantibodies are myositis-associated autoantibodies. Patients who met the 2017 ACR/EULAR classification criteria for DM, but were negative for DM-specific autoantibodies were also classified as DM[3].

Autoantibody testing was performed using one or more of the following techniques: ELISA, immunoprecipitation of proteins generated by *in vitro* transcription and translation (IVTT-IP), line blotting (EUROLINE myositis profile), or immunoprecipitation from 35S-methionine-labeled HeLa cell lysates.

Standard protocol approvals and patient consent

This study was approved by the Institutional Review Boards of the National Institutes of Health, the Johns Hopkins University, the Vall d'Hebron University Hospital, the Mayo Clinic, and the Charité-Universitätsmedizin Berlin. Written informed consent was obtained from each participant. All methods were performed in accordance with the relevant guidelines and regulations.

Gene Selection

A total of 338 genes of interest were chosen, including 200 cytokines, 131 cytokine receptors, and 7 immune checkpoints (Supplementary Table S1). Cytokines included all protein-coding genes in a set of HUGO Gene Nomenclature groups (chemokine ligands, interleukins, interleukin-6 family, interferons, and tumor necrosis factor superfamily), all genes with the UniProt molecular function keyword "cytokine", and four additional cytokines that did not meet these criteria (*CRLF1*, *IL22RA2*, *KITLG*, and *TXLNA*). Two cytokines identified from HUGO (*CCL3L1* and *CCL4L2*) that were not present in our reference genome (GRCh38) were excluded. Cytokine receptors included all protein-coding genes in a set of HUGO Gene Nomenclature groups (chemokine receptors, interleukin receptors, interferon receptors, and tumor necrosis factor receptor

superfamily), gene matches for the selected cytokines found in both of two receptor-ligand databases (Ramilowski 2015 & CellphoneDB v5.0.0), and three additional receptors that did not meet these criteria (*CRLF2*, *IL18BP*, *SIGIRR*)[4, 5]. Immune checkpoint genes included were *CD28*, *CD80*, *CD86*, *CD274*, *CTLA4*, *PDCD1*, and *PDCD1LG2*.

All cytokines and cytokine receptors were classified into families, including chemokines, interferons, interleukins, tumor necrosis factor (TNF), transforming growth factor-β (TGFB), and others (Supplementary Table S1). A subset of genes was also classified by the type of inflammation in which they are predominantly overexpressed, as defined by subsets of T cells, innate lymphoid cells (ILCs), and other interacting cells (Supplementary Table S2)[6-10]. These classifications were type 1 (mediated by *TBX21*+ ILC1 cells, T_H1 cells, and T_C1 cells), type 2 (mediated by *GATA3*+ ILC2 cells, T_H2 cells, and T_C2 cells), and type 3 (mediated by *RORC*+ ILC3 cells, T_H17 cells, and T_C17 cells)[6].

References for Supplementary Methods

1 Lloyd TE, Mammen AL, Amato AA, Weiss MD, Needham M, Greenberg SA. Evaluation and construction of diagnostic criteria for inclusion body myositis. Neurology 2014;83(5):426-33.
2 Casal-Dominguez M, Pinal-Fernandez I, Pak K, et al. Performance of the 2017 European Alliance of Associations for Rheumatology/American College of Rheumatology Classification Criteria for Idiopathic Inflammatory Myopathies in Patients With Myositis-Specific Autoantibodies. Arthritis Rheumatol 2022;74(3):508-17.
3 Lundberg IE, Tjarnlund A, Bottai M, et al. 2017 European League Against Rheumatism/American College of Rheumatology classification criteria for adult and juvenile idiopathic inflammatory myopathies and their major subgroups. Ann Rheum Dis 2017;76(12):1955-64.
4 Ramilowski JA, Goldberg T, Harshbarger J, et al. A draft network of ligand-receptor-mediated multicellular signalling in human. Nat Commun 2015;6:7866.
5 Vento-Tormo R, Efremova M, Botting RA, et al. Single-cell reconstruction of the early maternal-fetal interface in humans. Nature 2018;563(7731):347-53.
6 Annunziato F, Romagnani C, Romagnani S. The 3 major types of innate and adaptive cell-mediated effector immunity. J Allergy Clin Immunol 2015;135(3):626-35.

7 Sundrud MS, Trivigno C. Identity crisis of Th17 cells: many forms, many functions, many questions. Semin Immunol 2013;25(4):263-72.
8 Raphael I, Nalawade S, Eagar TN, Forsthuber TG. T cell subsets and their signature cytokines in autoimmune and inflammatory diseases. Cytokine 2015;74(1):5-17.
9 Gurram RK, Zhu J. Orchestration between ILC2s and Th2 cells in shaping type 2 immune responses. Cell Mol Immunol 2019;16(3):225-35.
10 Mills KHG. IL-17 and IL-17-producing cells in protection versus pathology. Nat Rev Immunol 2023;23(1):38-54.

Supplementary Table S1. List of genes included (n=338) by family.

Family	Cytokines	Receptors
Chemokines (and related)	CCL1, CCL2, CCL3, CCL3L3, CCL4, CCL4L2, CCL5, CCL7, CCL8, CCL11, CCL13, CCL14, CCL15, CCL16, CCL17, CCL18, CCL19, CCL20, CCL21, CCL22, CCL23, CCL24, CCL25, CCL26, CCL27, CCL28, CKLF, CMTM1, CMTM2, CMTM3, CMTM5, CMTM7, CMTM8, CX3CL1, CXCL1, CXCL2, CXCL3, CXCL5, CXCL6, CXCL8, CXCL9, CXCL10, CXCL11, CXCL12, CXCL13, CXCL14, CXCL16, CXCL17, PF4, PF4V1, PPBP, TAFA5, XCL1, XCL2	ACKR1, ACKR2, ACKR3, ACKR4, CCR1, CCR2, CCR3, CCR4, CCR5, CCR6, CCR7, CCR8, CCR9, CCR10, CCRL2, CX3CR1, CXCR1, CXCR2, CXCR3, CXCR4, CXCR5, CXCR6, HRH4, PITPNM3, XCR1
Interferons	IFNA1, IFNA2, IFNA4, IFNA5, IFNA6, IFNA7, IFNA8, IFNA10, IFNA13, IFNA14, IFNA16, IFNA17, IFNA21, IFNB1, IFNE, IFNG, IFNK, IFNL1, IFNL2, IFNL3, IFNW1	IFNAR1, IFNAR2, IFNGR1, IFNGR2, IFNLR1
Interleukins (and associated)	CLCF1, CNTF, CRLF1, CSF2, CTF1, C17ORF99, EBI3, IL1A, IL1B, IL1F10, IL1RN, IL2, IL3, IL4, IL5, IL6, IL7, IL9, IL10, IL11, IL12A, IL12B, IL13, IL15, IL16, IL17A, IL17B, IL17C, IL17D, IL17F, IL18, IL19, IL20, IL21, IL22, IL22RA2, IL23A, IL24, IL25, IL26, IL27, IL31, IL32, IL33, IL34, IL36A, IL36B, IL36G, IL36RN, IL37, LIF, OSM, TSLP, TXLNA	CNTFR, CRLF2, CSF2RA, CSF2RB, IL1R1, IL1R2, IL1RAP, IL1RAPL1, IL1RAPL2, IL1RL1, IL1RL2, IL2RA, IL2RB, IL2RG, IL3RA, IL4R, IL5RA, IL6R, IL6ST, IL7R, IL9R, IL10RA, IL10RB, IL11RA, IL12RB1, IL12RB2, IL13RA1, IL13RA2, IL15RA, IL17RA, IL17RB, IL17RC, IL17RD, IL17RE, IL18BP, IL18R1, IL18RAP, IL20RA, IL20RB, IL21R, IL22RA1, IL23R, IL27RA, IL31RA, LIFR, OSMR, SIGIRR
TNF	C1QTNF4, CD40LG, CD70, EDA, FASLG, LTA, LTB, TNF, TNFSF4, TNFSF8, TNFSF9, TNFSF10, TNFSF11, TNFSF12, TNFSF13, TNFSF13B, TNFSF14, TNFSF15, TNFSF18	CD27, CD40, EDA2R, EDAR, FAS, LTBR, NGFR, RELT, TNFRSF1A, TNFRSF1B, TNFRSF4, TNFRSF6B, TNFRSF8, TNFRSF9, TNFRSF10A, TNFRSF10B, TNFRSF10C, TNFRSF10D, TNFRSF11A, TNFRSF11B, TNFRSF12A, TNFRSF13B, TNFRSF13C, TNFRSF14, TNFRSF17, TNFRSF18, TNFRSF19, TNFRSF21, TNFRSF25
TGFB	BMP1, BMP2, BMP3, BMP4, BMP5, BMP6, BMP7, BMP8A, BMP8B, BMP10, BMP15, CER1, GDF1, GDF2, GDF3, GDF5, GDF6, GDF7, GDF9, GDF10, GDF11, GDF15, LEFTY1, LEFTY2, MSTN, NODAL, TGFB1, TGFB2, TGFB3	ACVR1, ACVR1B, ACVR1C, ACVR2A, ACVR2B, ACVRL1, BMPR1A, BMPR1B, BMPR2, ITGAV, ITGB6, ITGB8, TGFBR1, TGFBR2, TGFBR3
Other	AIMP1, ALKAL1, ALKAL2, AREG, CSF1, CSF3, FAM3B, FLT3LG, GPI, GPR15LG, GREM1, GREM2, GRN, KITLG, MIF, MSMP, NAMPT, SCGB3A1, SLURP1, SPP1, THNSL2, THPO, VSTM1	CSF1R, CSF3R, EGFR, FLT3, ITGA4, ITGA5, ITGA9, ITGB1, KIT, MPL
Immune Checkpoint	CD28, CD80, CD86, CD274, CTLA4, PDCD1, PDCD1LG2	

Supplementary Table S2. List of genes classified as type 1, type 2, or type 3. Only including genes predominantly associated with one of the three inflammation types. Excluding genes commonly involved in multiple inflammation types (e.g., IL2).

**Inflammation Type**	**Cytokines**	**Receptors**
ILC1/T_H1/T_C1-associated (Type 1)	CCL3, CCL4, CCL5, CXCL9, CXCL10, CXCL11, EBI3, IFNG, IL12A, IL18, IL27, LTA, TNF	CCR5, CXCR3, IFNGR1, IFNGR2, IL12RB2, IL18R1, IL18RAP, IL27RA, LTBR, TNFRSF1A, TNFRSF1B
ILC2/T_H2/T_C2-associated (Type 2)	AREG, CCL1, CCL11, CCL17, CCL22, CCL24, CCL26, IL1A, IL4, IL5, IL9, IL13, IL25, IL33, TNFSF4, TNFSF15, TSLP	CCR3, CCR4, CCR8, CRLF2, EGFR, IL1RL1, IL4R, IL5RA, IL9R, IL13RA1, IL17RB, TNFRSF4, TNFRSF25
ILC3/T_H17/T_C17-associated (Type 3)	CCL20, IL1B, IL6, IL17A, IL17F, IL22, IL23A, IL26	CCR6, IL6R, IL17RC, IL20RA, IL22RA1, IL23R

Supplementary Table S3. Adjusted p-values (q-values) of differential overexpression and log-fold change vs. all for type 1 genes in myositis groups. Values only included if logFC > 0.

Gene	DM		IMNM		ASyS		IBM		PM/Scl	
	**q-value**	**logFC**	**q-value**	**logFC**	**q-value**	**logFC**	**q-value**	**logFC**	**q-value**	**logFC**
CCL3	3.91E-02	0.545	9.58E-01	0.030	1.19E-02	0.990	1.35E-01	0.571	1.41E-01	0.966
CCL4	1.17E-01	0.373			1.49E-02	0.861	4.98E-07	1.426	3.13E-02	1.145
CCL5	7.04E-02	0.326			2.54E-01	0.425	8.15E-24	1.968	3.80E-03	1.113
CXCL10	8.04E-17	2.046			1.43E-02	1.105	5.42E-07	1.788	1.46E-02	1.591
CXCL11	4.11E-35	2.804			3.19E-02	1.011	3.38E-05	1.536	5.65E-02	1.313
CXCL9					9.85E-07	1.759	1.36E-21	2.951	3.48E-06	2.573
EBI3	4.92E-01	0.153			8.89E-03	0.799	9.00E-03	0.742	1.45E-04	1.565
IFNG					7.42E-02	0.857	4.61E-18	2.601	1.38E-02	1.501
IL12A	4.64E-05	0.610			8.34E-01	0.116	1.91E-02	0.525	8.04E-01	0.149
IL18	2.01E-15	0.808	6.43E-03	0.375	1.90E-01	0.309				
IL27	9.14E-10	1.180			8.43E-01	0.143	4.03E-04	1.016	7.52E-03	1.278
LTA	7.32E-01	0.078			1.96E-01	0.511	2.73E-07	1.283	1.92E-04	1.526
TNF	3.07E-01	0.181			1.70E-01	0.448	1.88E-06	1.027	7.95E-03	1.002
CCR5	9.60E-01	0.015			4.18E-01	0.462	4.73E-20	2.327	2.13E-03	1.534
CXCR3					6.31E-01	0.333	7.74E-23	2.474	5.01E-04	1.719
IFNGR1	2.23E-09	0.293	2.88E-04	0.220	8.78E-01	0.029				
IFNGR2	4.24E-03	0.153	2.86E-10	0.359	9.87E-01	0.004	2.41E-01	0.094		
IL12RB2	8.75E-15	0.920	3.82E-01	0.176					9.91E-01	0.008
IL18R1	3.90E-06	0.491			6.77E-01	0.142	2.84E-02	0.355	9.83E-01	0.012
IL18RAP	1.59E-02	0.428			3.54E-01	0.372	1.18E-02	0.631		
IL27RA					9.83E-01	0.008	8.09E-03	0.334	4.49E-04	0.676
LTBR	2.40E-01	0.072	7.01E-01	0.039					2.87E-01	0.176
TNFRSF1A	2.08E-10	0.359	7.21E-03	0.200	9.40E-01	0.019			4.76E-01	0.138
TNFRSF1B	2.30E-02	0.193			5.43E-01	0.136	9.41E-05	0.451	2.76E-02	0.452

Gene	Mi2		MDA5		NXP2		TIF1		HMGCR	
	**q-value**	**logFC**	**q-value**	**logFC**	**q-value**	**logFC**	**q-value**	**logFC**	**q-value**	**logFC**
CCL3	1.88E-02	1.290	9.96E-01	0.014			7.98E-01	0.233		
CCL4	2.66E-01	0.568	9.73E-01	0.082	8.90E-01	0.176	9.13E-01	0.099		
CCL5	5.02E-01	0.284	4.68E-01	0.840	9.93E-01	0.012	7.56E-01	0.187		
CXCL10	8.61E-03	1.600	2.98E-01	1.658	1.64E-04	2.040	2.63E-02	1.429		
CXCL11	2.23E-04	2.002	1.89E-03	2.600	4.27E-08	2.601	1.23E-07	2.352		
CXCL9										
EBI3	3.65E-01	0.419								
IFNG										
IL12A	4.37E-02	0.696			1.73E-01	0.559	9.47E-02	0.665		
IL18	2.58E-03	0.748	9.45E-01	0.077	5.52E-07	1.117	2.23E-03	0.737	1.77E-03	0.479
IL27	6.97E-04	1.513	3.46E-01	1.228	8.32E-02	0.873	2.77E-01	0.670		

LTA	4.87E-01	0.328					9.30E-01	0.070		
TNF	5.23E-01	0.254			9.65E-01	0.052	9.11E-01	0.073		
CCR5	7.76E-01	0.180								
CXCR3	7.63E-01	0.188	9.88E-01	0.035						
IFNGR1	4.39E-03	0.335	6.90E-01	0.196	1.41E-02	0.306	1.34E-01	0.214	1.47E-02	0.181
IFNGR2	1.92E-02	0.285			6.29E-01	0.096	4.64E-01	0.119	7.68E-08	0.355
IL12RB2	2.37E-02	0.684	4.22E-01	0.699	3.30E-06	1.225	8.76E-02	0.588	5.38E-01	0.158
IL18R1	7.66E-02	0.437			2.18E-01	0.384	3.02E-02	0.569		
IL18RAP	3.98E-01	0.345	9.75E-01	0.056	7.71E-01	0.226	4.74E-01	0.384		
IL27RA			8.92E-01	0.109						
LTBR	7.51E-01	0.048					6.41E-01	0.087	6.28E-01	0.057
TNFRSF1A	6.67E-04	0.447	4.29E-01	0.329	4.99E-03	0.391	2.29E-01	0.214	1.82E-02	0.206
TNFRSF1B	1.27E-01	0.280	8.96E-01	0.101			5.53E-01	0.155		

Gene	**SRP**		**Jo1**	
	**q-value**	**logFC**	**q-value**	**logFC**
CCL3	9.71E-01	0.149	1.97E-04	1.582
CCL4			9.16E-05	1.457
CCL5			1.98E-03	0.995
CXCL10			1.71E-06	2.096
CXCL11			1.08E-05	1.962
CXCL9			2.33E-15	2.982
EBI3			1.72E-02	0.935
IFNG			3.13E-04	1.680
IL12A			5.77E-02	0.623
IL18	1.00E+00	0.000	3.64E-02	0.509
IL27			2.80E-01	0.537
LTA			5.92E-02	0.776
TNF			1.01E-01	0.591
CCR5			3.59E-02	0.996
CXCR3			5.84E-02	0.924
IFNGR1	1.87E-01	0.298	4.54E-01	0.102
IFNGR2	1.70E-01	0.306	3.24E-01	0.130
IL12RB2	9.17E-01	0.200		
IL18R1			1.96E-01	0.340
IL18RAP			3.53E-01	0.402
IL27RA			8.38E-01	0.054
LTBR				
TNFRSF1A	8.08E-01	0.146	2.72E-01	0.166
TNFRSF1B			6.55E-02	0.331

Supplementary Table S4. Adjusted p-values (q-values) of differential overexpression and log-fold change vs. all for type 2 genes in myositis groups. Values only included if logFC > 0.

Gene	DM		IMNM		ASyS		IBM		PM/Scl	
	q-value	logFC	q-value	logFC	q-value	logFC	q-value	logFC	q-value	logFC
AREG	1.15E-11	1.522	7.76E-01	0.137			7.71E-01	0.127		
CCL1					4.48E-01	0.231	4.50E-04	0.639	5.57E-01	0.246
CCL11	2.52E-15	1.775	8.11E-01	0.115	1.33E-02	1.001	3.03E-01	0.391	2.78E-01	0.761
CCL17			3.67E-01	0.288			4.67E-09	1.667	6.63E-01	0.324
CCL22			9.62E-01	0.023			2.09E-07	1.460	4.68E-02	1.049
CCL24							8.88E-03	0.768	7.11E-01	0.273
CCL26	9.24E-01	0.024	5.90E-01	0.177			1.08E-04	1.039		
IL13	6.81E-01	0.084					2.45E-02	0.573		
IL1A					1.62E-04	0.877	8.30E-03	0.591	4.87E-01	0.353
IL25			7.53E-01	0.080			4.19E-02	0.434		
IL33			1.02E-05	0.546	2.46E-01	0.277	2.96E-01	0.179	8.30E-01	0.098
IL4										
IL5					7.20E-01	0.152	1.80E-01	0.261	1.46E-01	0.582
IL9										
TNFSF15	1.60E-01	0.208			3.85E-01	0.292	1.30E-04	0.746	4.83E-02	0.670
TNFSF4	2.06E-01	0.180			5.49E-02	0.453	2.01E-03	0.588	2.58E-01	0.425
TSLP	4.34E-02	0.216	8.49E-05	0.458			9.57E-01	0.010		
CCR3							2.88E-01	0.349		
CCR4					8.73E-01	0.126	2.42E-09	1.568	3.08E-04	1.585
CCR8					6.54E-01	0.224	6.58E-09	1.459	2.23E-02	1.113
CRLF2	9.72E-01	0.008			7.81E-01	0.170	4.54E-01	0.205	1.35E-02	1.153
EGFR			9.83E-01	0.004			7.42E-01	0.042		
IL13RA1			5.08E-07	0.241	6.70E-01	0.056	8.94E-01	0.010		
IL17RB	9.64E-01	0.006	9.81E-01	0.006						
IL1RL1	6.14E-08	1.029			5.17E-02	0.691				
IL4R	7.35E-01	0.037			4.06E-01	0.186	5.03E-02	0.269	1.51E-03	0.673
IL5RA							1.65E-12	1.821	2.30E-01	0.695
IL9R							1.17E-02	0.740		
TNFRSF25					2.29E-01	0.251	1.33E-02	0.357	1.81E-08	1.124
TNFRSF4	4.80E-02	0.298			9.68E-01	0.025			1.04E-07	1.533

Gene	Mi2		MDA5		NXP2		TIF1		HMGCR	
	q-value	logFC	q-value	logFC	q-value	logFC	q-value	logFC	q-value	logFC
AREG	4.84E-03	1.485	9.03E-01	0.302	1.93E-03	1.603	5.32E-03	1.444	9.49E-01	0.048
CCL1										
CCL11	4.17E-05	1.994	8.80E-01	0.393	2.82E-02	1.264	5.75E-03	1.494	9.62E-01	0.036
CCL17									1.13E-01	0.529
CCL22									7.27E-01	0.169

CCL24										
CCL26	1.26E-01	0.668	7.38E-01	0.645					3.88E-01	0.297
IL13			8.08E-01	0.493	9.02E-01	0.122	8.35E-01	0.134		
IL1A			8.95E-01	0.198						
IL25			9.99E-01	0.002					4.72E-01	0.191
IL33									5.68E-04	0.504
IL4										
IL5										
IL9										
TNFSF15			6.57E-01	0.543	8.29E-01	0.144	8.61E-01	0.092		
TNFSF4	8.56E-01	0.067					7.38E-01	0.151	9.07E-01	0.041
TSLP	4.13E-01	0.200			3.40E-01	0.303	9.49E-01	0.028	5.83E-05	0.530
CCR3					9.59E-01	0.082	9.10E-01	0.098		
CCR4					9.62E-01	0.073				
CCR8										
CRLF2			6.06E-01	0.854	2.99E-01	0.585				
EGFR									9.79E-01	0.006
IL13RA1									3.28E-05	0.232
IL17RB	8.23E-01	0.067	6.60E-01	0.443	9.48E-01	0.050				
IL1RL1	5.14E-02	0.891			1.19E-04	1.530	2.27E-01	0.703		
IL4R	5.98E-01	0.122					7.14E-01	0.113		
IL5RA										
IL9R										
TNFRSF25										
TNFRSF4	2.56E-01	0.377	7.48E-01	0.463			6.78E-01	0.199		

Gene	SRP		Jo1	
	**q-value**	**logFC**	**q-value**	**logFC**
AREG	9.18E-01	0.379	5.11E-01	0.442
CCL1			1.04E-01	0.449
CCL11	9.24E-01	0.348	2.13E-03	1.447
CCL17				
CCL22				
CCL24	9.93E-01	0.032		
CCL26				
IL13				
IL1A			3.78E-02	0.700
IL25				
IL33	2.51E-01	0.574	1.15E-01	0.397
IL4				
IL5	9.69E-01	0.073	8.03E-01	0.093
IL9				
TNFSF15			8.50E-03	0.729
TNFSF4			1.13E-03	0.804

TSLP	9.11E-01	0.162		
CCR3				
CCR4			9.18E-01	0.072
CCR8			4.04E-01	0.355
CRLF2	9.23E-01	0.256	8.57E-01	0.097
EGFR				
IL13RA1	2.69E-01	0.222	5.14E-01	0.074
IL17RB	9.53E-01	0.105		
IL1RL1	9.89E-01	0.053	7.37E-05	1.363
IL4R	8.57E-01	0.191	6.18E-02	0.374
IL5RA				
IL9R				
TNFRSF25	9.58E-01	0.079	1.51E-01	0.328
TNFRSF4				

Supplementary Table S5. Adjusted p-values (q-values) of differential overexpression and log-fold change vs. all for type 3 genes in myositis groups. Values only included if logFC > 0.

Gene	DM		IMNM		ASyS		IBM		PM/Scl	
	**q-value**	**logFC**	**q-value**	**logFC**	**q-value**	**logFC**	**q-value**	**logFC**	**q-value**	**logFC**
CCL20	3.29E-02	0.500	4.37E-01	0.264	6.70E-06	1.370	5.81E-01	0.184	1.48E-01	0.871
IL17A			6.48E-01	0.095			2.79E-02	0.406	5.36E-01	0.262
IL17F			7.78E-01	0.055	8.26E-01	0.082	4.80E-03	0.449	2.44E-01	0.390
IL1B			6.40E-02	0.495	2.05E-03	0.937	6.75E-01	0.144	4.64E-01	0.471
IL22			8.24E-01	0.060	8.10E-01	0.121	2.95E-01	0.224	2.09E-01	0.576
IL23A	1.66E-01	0.237			2.27E-01	0.412	5.94E-02	0.444	1.66E-01	0.594
IL26										
IL6	1.79E-01	0.374	4.91E-01	0.290	5.59E-01	0.421			5.12E-01	0.541
CCR6	6.79E-02	0.563			3.68E-01	0.621	2.33E-01	0.529	1.62E-01	1.060
IL17RC									9.79E-01	0.009
IL20RA			4.30E-04	0.580	8.79E-01	0.082	1.10E-01	0.343		
IL22RA1	1.23E-15	1.260	3.38E-02	0.473			1.33E-01	0.398	9.65E-01	0.038
IL23R							1.31E-02	0.651	1.18E-01	0.778
IL6R					9.35E-01	0.030				

Gene	Mi2		MDA5		NXP2		TIF1		HMGCR	
	**q-value**	**logFC**	**q-value**	**logFC**	**q-value**	**logFC**	**q-value**	**logFC**	**q-value**	**logFC**
CCL20	6.74E-03	1.425			8.61E-01	0.201	7.97E-01	0.201		
IL17A			9.22E-01	0.116					6.05E-01	0.127
IL17F					9.61E-01	0.039			8.66E-01	0.044
IL1B	5.36E-01	0.318							1.76E-01	0.445
IL22					4.06E-01	0.426			9.20E-01	0.039

IL23A					3.00E-01	0.479	8.38E-01	0.120	9.17E-01	0.045
IL26										
IL6	1.19E-01	0.869	8.88E-01	0.351	9.85E-01	0.039	9.50E-01	0.071	6.36E-01	0.256
CCR6	6.33E-01	0.364	3.56E-01	1.629	5.36E-01	0.617				
IL17RC			8.83E-01	0.099			7.61E-01	0.069		
IL20RA	1.41E-01	0.468							8.36E-03	0.519
IL22RA1	1.24E-02	0.996	3.09E-01	1.072	9.59E-07	1.663	4.28E-02	0.880	4.29E-02	0.522
IL23R										
IL6R			7.95E-01	0.264						

**Gene**	**SRP**		**Jo1**	
	**q-value**	**logFC**	**q-value**	**logFC**
CCL20	4.29E-01	0.957	4.89E-06	1.786
IL17A			9.59E-01	0.020
IL17F	9.61E-01	0.074	3.94E-01	0.227
IL1B	7.49E-01	0.557	1.22E-02	1.023
IL22	9.58E-01	0.109	7.44E-01	0.130
IL23A			3.31E-02	0.723
IL26				
IL6	9.29E-01	0.340	5.50E-02	1.031
CCR6			8.36E-02	1.100
IL17RC				
IL20RA	3.47E-01	0.655	2.65E-01	0.395
IL22RA1	9.27E-01	0.238		
IL23R				
IL6R				

Supplementary Figure S1. Venn Diagrams of differentially overexpressed genes. (A) Venn Diagram of differentially overexpressed genes for the 5 myositis clinical groups (DM, IMNM, ASyS, IBM, PM/Scl). For each clinical group, all genes differentially overexpressed for the clinical group or for any of its autoantibody subgroups (e.g., HMGCR and SRP for IMNM) were included in the diagram. (B) Venn Diagram of differentially overexpressed genes for DM and its autoantibody subgroups (Mi2, MDA5, NXP2, and TIF1), excluding genes differentially overexpressed in any other group.

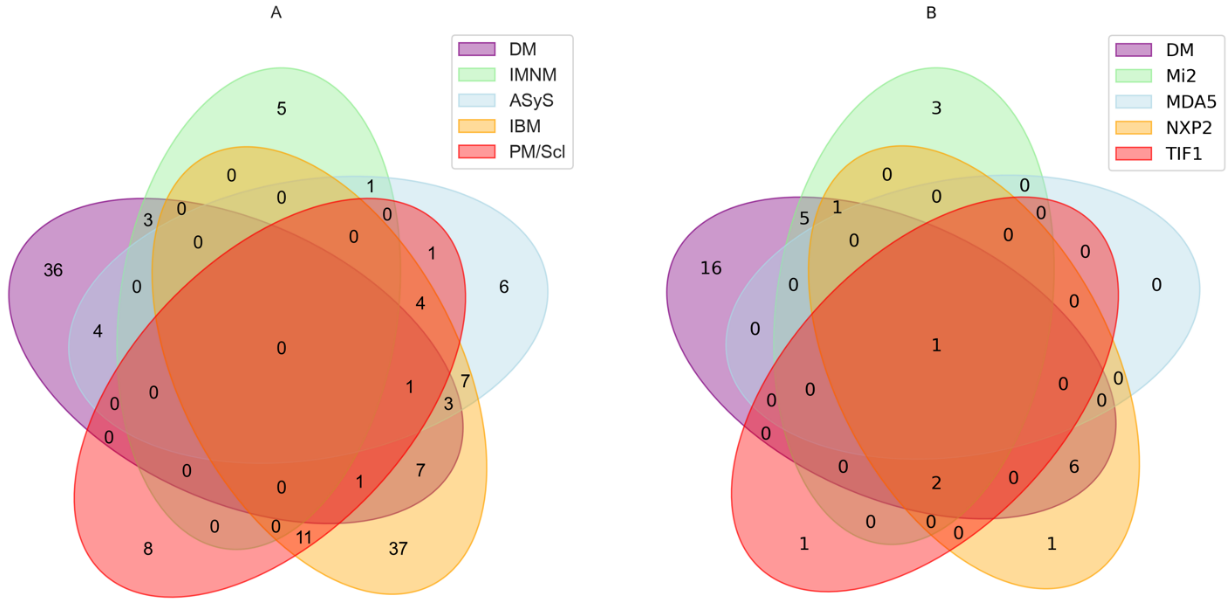

Supplementary Figure S2. Boxplots of top differentially overexpressed genes by clinical group. Showing expression of top 2 group-specific differentially overexpressed genes for each clinical group: DM (*TNFSF10*, *GDF15*), IMNM (*IL17B*, *IL13RA1*), ASyS (*CXCL8*, *CCL20*), IBM (*CCL5*, *CCR5*), PM/Scl (*TNFRSF25*, *TNFRSF4*), and NT (*AIMP1*, *MSTN*).

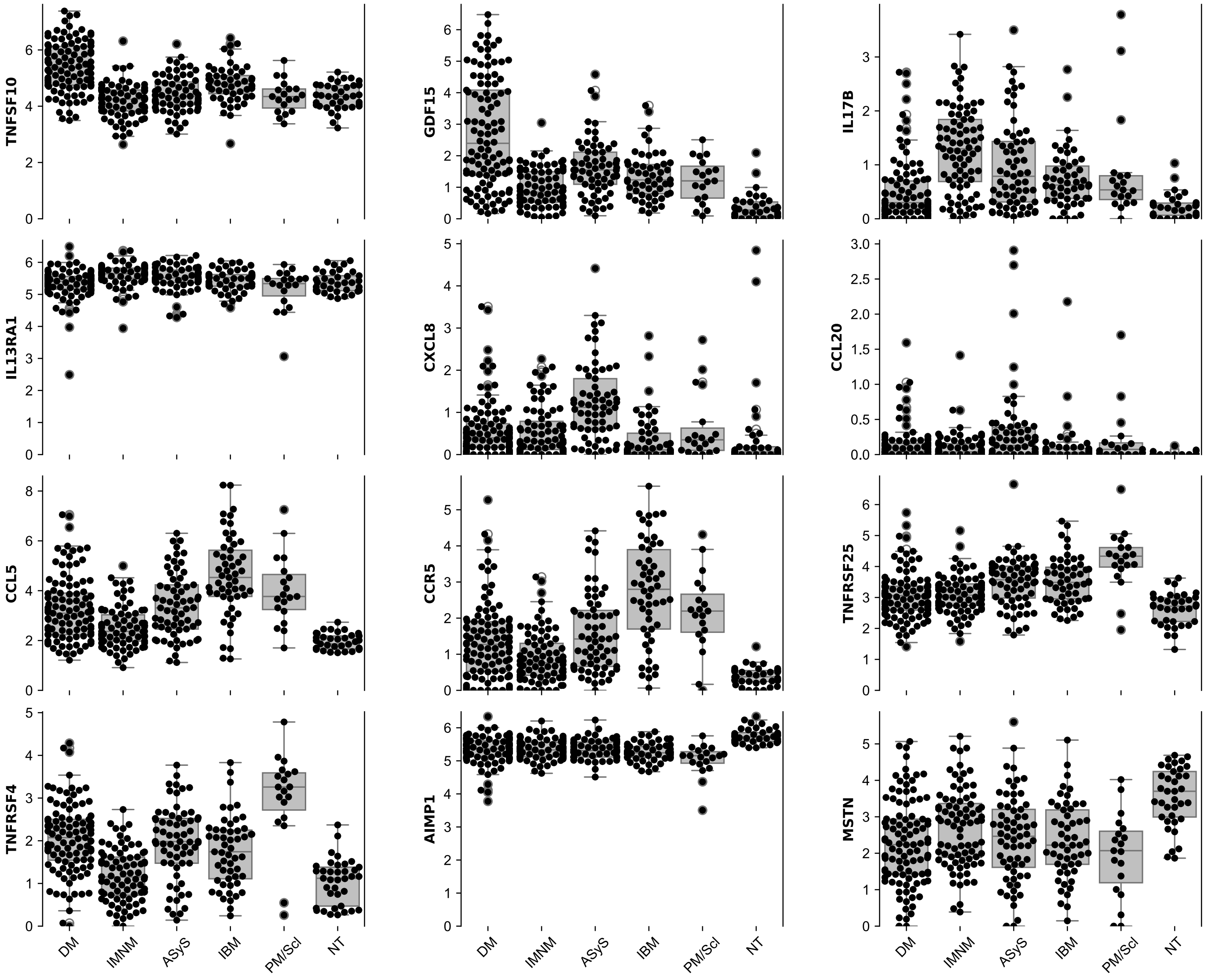

Supplementary Figure S3. Boxplots of top differentially overexpressed genes by autoantibody subgroup. Showing expression of top 2 group-specific differentially overexpressed genes for each autoantibody subgroup: Mi2 (*IL11*, *GRN*), NXP2 (*IFNB1*, *IL15RA*), TIF1 (*IL31RA*), HMGCR (*IL17B*, *IL10RB*), Jo1 (CXCL8, *CCL20*). There was only 1 TIF1-specific differentially overexpressed gene, and 0 MDA5- or SRP-specific differentially overexpressed genes.

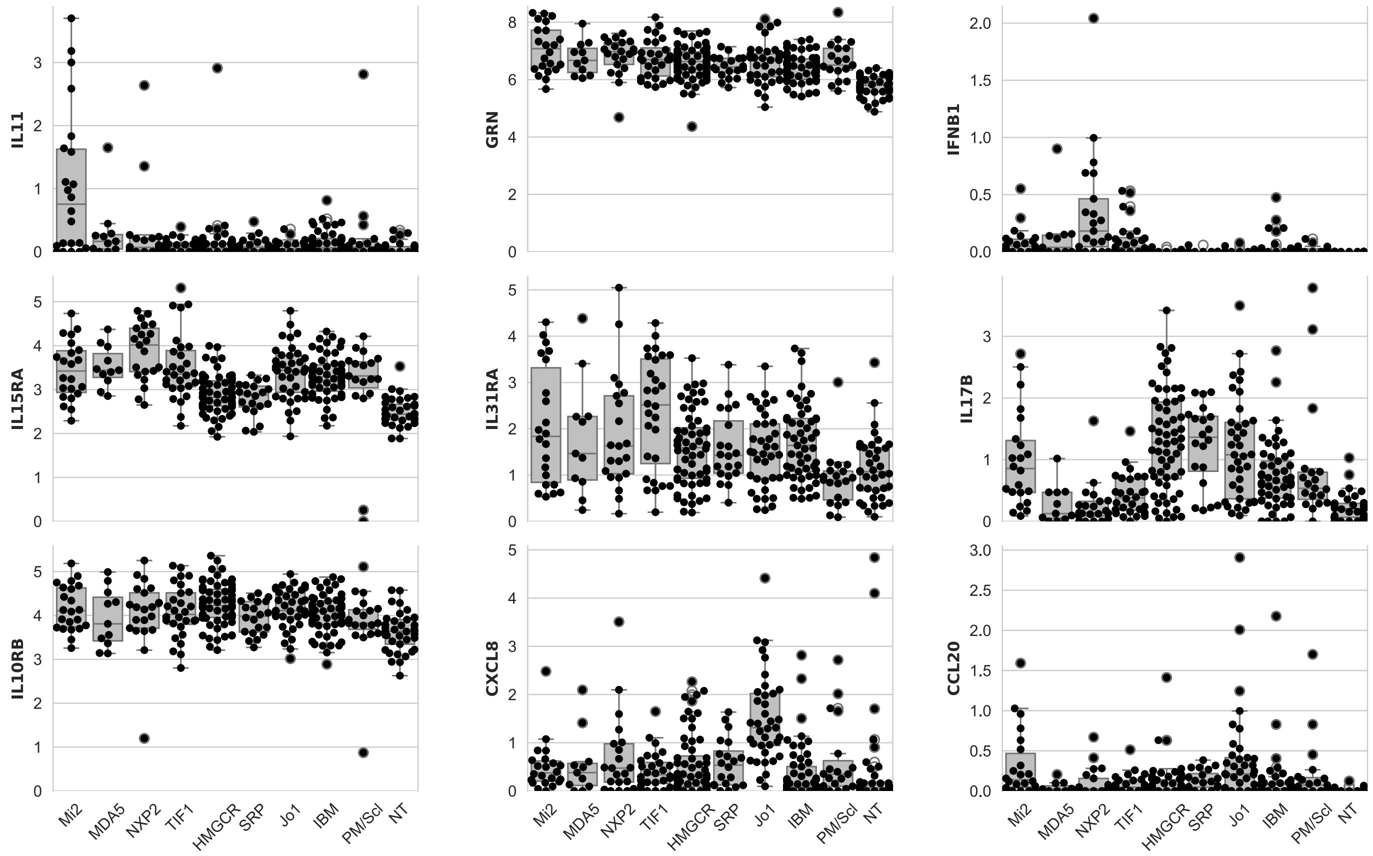

Supplement: Supplement 1 [file media-1.pdf]
